# Jidangga-7 ameliorates non-small cell lung cancer by regulating gut microbiota function

**DOI:** 10.3389/fmicb.2025.1516685

**Published:** 2025-03-05

**Authors:** Changcheng Yue, Chula San, Shichao Deng, Jingjing Wang, Xueying Shen, Hongqing Wang, Liyan Huang, Renbatu Bu, Dong Wang

**Affiliations:** ^1^Affiliated Hospital of Inner Mongolia Minzu University, Tongliao, Inner Mongolia, China; ^2^Clinical Research Centre for Malignant Tumours of Mongolian Medicine in Inner Mongolia Autonomous Region, Tongliao, China

**Keywords:** non-small cell carcinoma, gut microbiota, Jidangga-7, 16S rRNA, broadly targeted metabolomics

## Abstract

**Objective:**

This study aims to assess the effects of Jidangga-7 on enhancing gut microbiota function in non-small cell lung cancer.

**Materials and methods:**

Eighteen mice were screened and randomly divided into three groups: a control group, a model group with induced non-small cell lung cancer, and a treatment group receiving Jidangga-7. A549 tumor cells were implanted in the mice, and tumor formation was monitored. Upon successful tumor induction, the treatment group received Jidangga-7 via oral gavage, while the other groups received an equivalent volume of saline. After the final dose, intestinal tissues were collected from each group, and microbial amplicon 16S analysis and non-extensive targeted metabolomics were employed to characterize intestinal fiber and associated metabolites.

**Results:**

By quantifying the contribution of individual species to the variations between the groups, the Sipmer results highlighted the top 10 species and their abundance that contribute to the differences between the two groups. Specifically, Jidangga-7 demonstrated a regulatory effect on various taxa such as Gammaproteobacteria, Bacilli, and Desulfovovoviridae. At the family level, administration of Jidangga-7 exhibited a regulatory effect on families including *Desulfovibrionaceae*, *Lachnospiraceae*, and *Eggerthellaceae*, compared to the model group. In untargeted metabolomics analyses, principal component analysis effectively differentiated the groups from one another. Subsequently, metabolites with a variable importance in projection score > 1 were screened. The Kyoto Encyclopedia of Genes and Genomes pathway analysis revealed 20 metabolite pathways, encompassing metabolism of cofactors and vitamins, bacterial metabolism, antimicrobial pathways, and xenobiotics biodegradation and metabolism.

**Conclusion:**

Jidangga-7 exerted a positive influence on the intestinal microbial environment in mice with non-small cell carcinoma, ameliorating the dysbiosis induced by non- small cell lung cancer. This intervention inhibited the growth of pathogenic bacteria while fostering the growth of beneficial strains.

## Graphical abstract

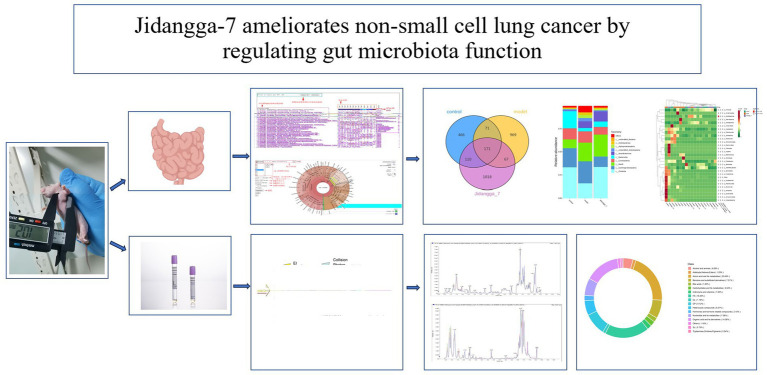

## 1 Introduction

According to global cancer statistics for 2021, lung cancer stands as the foremost cause of cancer-related mortality worldwide, with its incidence steadily increasing over recent decades (Sung et al., 2021). It has become one of the most prevalent malignancies globally and remains a leading cause of death from malignant tumors annually (The Lancet, 2019; Gao et al., 2020; Thai et al., 2021). Survey data from 2020 indicate approximately 2.2 million new cases of lung cancer and 1.8 million deaths worldwide, with the disease’s mortality rate ranking the highest in the world (Sung et al., 2021). Lung cancer is broadly classified into non-small cell lung cancer (NSCLC) and small cell lung cancer (SCLC) based on histological types, with NSCLC comprising approximately 85% of primary lung cancer cases. Further, NSCLC exhibits notable gender and pathological type differences, with lung adenocarcinoma being the predominant subtype, accounting for over 40% of all lung cancers and demonstrating an upward trend in incidence, especially among women (Zappa and Mousa, 2016; Barta et al., 2019; Szalontai et al., 2021; Nicholson et al., 2022). This trend is more pronounced in postmenopausal women, peaking around the age of 80 (Elbasheer et al., 2024). Recent data (Szalontai et al., 2021) show an overall 5-year survival rate of only about 15% for NSCLC. Treatment strategies vary based on histological types, with platinum-containing two-agent chemotherapy regimens remaining among the main options for advanced NSCLC. However, resistance to epidermal growth factor receptor-tyrosine kinase inhibitors (EGFR-TKIs) has emerged as a poignant factor contributing to treatment failure in NSCLC. Despite their efficacy, platinum-based drugs face limitations in clinical application due to chronic toxic side effects and drug resistance issues. The development of drugs targeting this aspect in clinical settings remains limited, presenting a substantial challenge in extending patient survival. Hence, there is a critical need to make rational choices to maintain drug efficacy while reducing toxic side effects in lung cancer treatment (Elbasheer et al., 2024).

Chinese medicine represents a revered asset of Chinese civilization, embodying over 5,000 years of cultural heritage and playing a significant impact on public health worldwide. Jidangga-7, clinically applied for the treatment of parasitic infections, demonstrates precise clinical efficacy. Comprised of ingredients such as garlic (*Allium sativum* L.), Physalis peruviana [*Embelia laeta* (L.) Mez], purple rivulet (*Butea monosperma*), *Chrysanthemum coronarium* (Semen Abutili), schizonepeta (*Nepeta cataria* L.), tarragon (*Artemisia gmelinii* Web. ex Stechm.), Chinese Iris Seed [*Iris lactea* Pall.var.chinensis (Flsch.) Koidz.], and other Mongolian medicinal herbs, Jidangga-7 primarily targets the eradication of parasites and management of gastrointestinal worm diseases (Yu tuo·yundangongbu, 1991; Isibaljuur, 2015; Song and Muren, 2015; San, 2024). A large number of studies have shown that Purple Rivet can inhibit the proliferation mechanism of lung cancer cells (Di et al., 2019; Zhang et al., 2019), induce the apoptosis mechanism of lung cancer cells (Jung et al., 2015) and antagonize the drug-resistance mechanism of lung cancer cells (Jung et al., 2015) thorns belongs to the plants with dual use of medicine and food, and it is confirmed by experiments that the volatile oil extract of thorns has killing and anti-tumor effects on human lung cancer A549 (Zang et al., 2006); malus seeds can improve the complications such as pleural heat stress pain in lung cancer patients, and it can lead to an increase in the survival rate (Jia and Yang, 2000; Liang et al., 2001).

The human gut harbors a vast and diverse microbial community known as gut flora, comprising approximately 10^13^–10^14^ microorganisms and over 1,000 species of bacteria (Hong et al., 2021). This intestinal microbiota undergoes development from birth, with notable changes occurring predominantly before the age of three, gradually increasing in complexity and diversity until reaching a stable equilibrium in adulthood (Bhatt et al., 2017). In most healthy individuals, the composition of microbiota is extremely similar, with over 90% of the bacteria belonging to the phyla thick-walled and mycobacterium, followed by *Micrococcus*, *Aspergillus*, and *Actinobacteria*, collectively constituting 99% of the microbiota. The “core” microbiota consists of approximately 60 species, mainly including *Bifidobacteria*, *Eubacteria*, *Clostridia*, *Faecalibacterium*, and *Ruminococcus* (Bingula et al., 2017). Typically, the intestinal flora maintains a delicate balance, with bacteria regulating each other and relying on mutual interactions to sustain a microecological equilibrium in terms of both quality and quantity. Under normal conditions, the intestinal flora and the host are closely linked through metabolic- immune- neuroendocrine networks, establishing a mutually beneficial symbiotic relationship (Kho and Lal, 2018).

Gut microbiota dysbiosis has been implicated in the development and progression of various lung diseases. Research indicates that certain dietary components, such as antioxidants and phytoestrogens, can inhibit the onset of lung cancer by modulating the gut microbiota (Caesar et al., 2015; Qadir and Cheema, 2017). Consequently, the gut microbiota represents a potential target for pharmacological interventions. Moreover, understanding the interplay between traditional Chinese medicine (TCM), combination therapies, and the gut microbiota could offer valuable insights into treatment mechanisms (Lv et al., 2019; Zhu et al., 2020). In recent studies, the combination of gut microbiota analysis and broadly targeted metabolomics, including 16S rRNA sequencing, has provided reliable technological support for unraveling the mechanisms underlying the treatment of cancer (Song et al., 2018). In this study, we investigated the impact of Jidangga-7, an aqueous extract obtained by boiling, on the intestinal flora in a rat model of NSCLC using 16S rRNA sequencing and broadly targeted metabolomics as the entry point. Our aim was to uncover potential evidence of dysbiosis and altered metabolites within the characteristic gut microflora associated with NSCLC, with the ultimate goal of identifying novel research directions for disease treatment.

## 2 Materials and methods

### 2.1 Drugs and reagents

Jidangga-7 (M20201000000) was sourced from the Affiliated Hospital of Inner Mongolia Minzu University. High glucose Dulbecco’s modified Eagle medium (DMEM) (Thermo Fisher SCIENTIFIC), fetal bovine serum (FBS) (Thermo Fisher SCIENTIFIC), phosphate- buffered saline (PBS) (Thermo Fisher SCIENTIFIC), and 0.25% trypsin and other reagents and chemicals were purchased from commercial suppliers (Thermo Fisher SCIENTIFIC).

### 2.2 Instruments and equipment

Carbon dioxide incubator (NU-5700, Nuarire), inverted biomicroscope (DM IL LED, Leica), biomicroscope (DM3000, Leica), low-speed centrifuge (LC-4012, Anhui Zhongke Zhongjia Scientific Instrument Co., Ltd.), electronic analytical balance (XJ620M, Shanghai Tianmei Balance Instrument Co., Ltd.), fully automated Tissue dehydrator (Thermo Excelsior ES), embedding machine (Thermo HistoStar), rotary paraffin slicer (Thermo HM 340E), slicer (Thermo SLIMLINE HOTPLATE 230 V), small high-speed refrigerated centrifuge (KH20R Anhui Zhongke Zhongjia Scientific Instrument Co., Ltd.).

### 2.3 Animal culture

Animal experiments were conducted in accordance with the guidelines set by the Experimental Animal Ethics Committee of the Affiliated Hospital of Inner Mongolia Minzu University, No. NM-LL-2024-03-15-01. The study protocol adhered to the National Institute of Health Guidelines for the Ethical Use of Animals. Five- week-old male Balb/c nude mice were purchased from the Changsheng Experimental Animal Centre of Liaoning Province, China. They were subsequently housed in the specific pathogen free (SPF)-grade experimental animal facility at Inner Mongolia Minzu University. The mice were maintained under controlled conditions, with a temperature of 23 ± 1°C, humidity of 55 ± 5%, and a 12-h light-dark cycle. They were provided ad libitum access to food, water, and physical activities throughout the study period.

### 2.4 Cell culture

A549 cells were obtained from the Cell Bank of the Chinese Academy of Sciences (Shanghai, China) (ZQ0003). They were cultured in DMEM supplemented with 1%(v/v) antibiotic-antimycotic solution (100 U/mL penicillin and 100 U/mL streptomycin) and 10%(v/v) FBS at 37°C in a humidified atmosphere containing 5% carbon dioxide (CO₂).

### 2.5 Cell culture, animal modeling, and grouping

The mice were allowed to acclimatize to the feeding environment for 1 week. A549 cells in logarithmic growth phase, reaching approximately 80% confluence, were collected and counted manually using a cell counter plate. The cells were then resuspended in PBS to achieve a density of 1 × 10^7^/100 μL, and maintained on ice until further use. For inoculation, the skin in the middle and posterior part of the right axilla of the mice was prepared and disinfected using 75% ethanol. Subsequently, 100 μL of the cell suspension was injected subcutaneously into the specified area using a 1 mL syringe. Following injection, the needle was slowly withdrawn, and gentle pressure was applied to the injection site with a cotton swab for 30 s to prevent extravasation of the cell suspension (San, 2024). Tumor volume was calculated, and modeling was successful when the average tumor volume of the model group reached 130 mm^3^. The formula was applied: V (mm^3^) = Dd^2^/2 (D is the long diameter of the tumor, d is the short diameter of the tumor). Tumor size was measured every other day using digital vernier calipers (Hou, 2022). Subsequently, the mice were randomly divided into two groups of 10 mice each. Following statistical analysis to assess differences in body weight and tumor size between groups, the mice were numbered and then housed in cages accordingly. Jidangga-7 was administered for 3 weeks according to the adult conversion dose (Huang et al., 2004), after 3 weeks mice were anaesthetized using isoflurane, blood was taken from the abdominal aorta, centrifuged and serum was stored in a −80°C refrigerator to be tested. Intestinal samples taken at and below the end of the mouse ileum were placed in sterile Eppendorf tubes, snap-frozen with liquid nitrogen, and then kept in a −80°C biofreezer for further analysis after the abdominal cavity was opened and adjusted to the position of the ileocecal valve.

### 2.6 Sequencing

Total genomic DNA was extracted from intestinal samples from mice (*n* = 6 per group) with lung cancer using the cetyltrimethylammonium bromide method. Subsequently, the concentration and purity of the extracted DNA were assessed using 1% agarose gel electrophoresis. The DNA was diluted to a final concentration of 1 ng/μL using sterile water. The amplification of distinct regions of the 16S rRNA was performed using specific primers with attached barcodes. Each polymerase chain reaction (PCR) reactions were carried out with 15 μL of Phusion^®^ High-Fidelity PCR Master Mix, 2 μM of forward and reverse primers (see in Table 1), and approximately 10 ng template DNA. The thermal cycling protocol consisted of initial denaturation step at 98°C for 1 min, followed by 30 cycles of denaturation at 98°C for 10 s, annealing at 50°C for 30 s, and elongation at 72°C for 30 s. A final extension step was carried out at 72°C for 5 min. An equal volume of 1X loading buffer containing SYBR Green was mixed with the PCR products. Subsequently, electrophoresis was conducted on a 2% agarose gel for detection. To ensure uniform representation, PCR products were mixed in equidensity ratios. The resultant mixture of PCR products was then purified using the Qiagen Gel Extraction Kit (Qiagen, Germany). Sequencing libraries were generated using TruSeq^®^ DNA PCR-Free Sample Preparation Kit, following the manufacturer’s instructions, and index codes were added as per standard protocols. The library quality was assessed using the Qubit@ 2.0 Fluorometer and the Agilent Bioanalyzer 2100 system. Finally, the libraries were sequenced on an Illumina NovaSeq platform, generating 250 base pair (bp) paired- end reads.

**Table 1:** Sequence details.

Types	Amplified region	Fragment length	Primers	Sequences (5′–3′)
Bacterial 16 s	V4	300 bp	515F	GTGCCAGCMGCCGCGGTAA
			806R	GGACTACHVGGGTWTCTAAT

### 2.7 Sample preparation and extraction

#### 2.7.1 Liquid samples class I

The serum sample (*n* = 6 per group), stored at −80°C, was thawed on ice and vortexed for 10 s. The next step involved adding 50 μL of the sample and 300 μL of extraction solution (ACN: methanol = 1:4, V/V) containing internal standards to a 2 mL microcentrifuge tube. The mixture was vortexed for 3 min and then centrifuged at 12,000 rpm for 10 min at 4°C. Following centrifugation, a 200 μL of the supernatant was collected and placed in a −20°C freezer for 30 min. It was then centrifuged at 12,000 rpm for 3 min at 4°C. Finally, a 180 μL aliquots of the supernatant were transferred for liquid chromatography- mass spectrometry (LC–MS) analysis.

#### 2.7.2 T3 ultra-performance liquid chromatography conditions

The sample extracts were analyzed using an LC-ESI-MS/MS system (ExionLC AD;^1^ MS, QTRAP^®^ System).^2^ The analytical conditions were as follows: for UPLC, the column utilized was the Waters Acquity UPLC HSS T3 C18 (1.8 μm, 2.1 mm × 100 mm) operated at a temperature of 40°C. The flow rate was set at 0.4 mL/ min, with an injection volume of 2 μL. The solvent system consisted of water (0.1% formic acid) and acetonitrile (0.1% formic acid), with a gradient program for solvent B: starting at 5% and increasing to 20% over 2 min, further increasing to 60% over the subsequent 3 min, then to 99% in 1 min and held for 90 s, followed by a return to 5% within 6 s and 144 s, respectively.

#### 2.7.3 ESI-QTRAP-MS/MS

Linear ion trap (LIT) and triple quadrupole (QQQ) scans were acquired using a triple quadrupole- linear ion trap mass spectrometer (QTRAP), specifically the QTRAP^®^ LC–MS/MS System. This system was equipped with an electrospray ionization (ESI) turbo ion-spray interface and operated in both positive and negative ion modes. The instrument was controlled by Analyst 1.6.3 software (Sciex). The operating parameters for the ESI source were as follows: the source temperature was set to 500°C, the ion spray voltage (IS) was 5,500\V in positive mode and −4500 V in negative mode. Additionally, the ion source gas I(GSI), gas II(GSII), and curtain gas (CUR) were maintained at pressures of 55, 60, and 25.0 psi, respectively. The collision gas (CAD) was set to high. Instrument tuning and mass calibration were performed using 10 and 100 μmol/L polypropylene glycol solutions in QQQ and LIT modes, respectively. During data acquisition, a specific set of multiple reaction monitoring (MRM) transitions were monitored for each period based on the metabolites eluted during that period.

### 2.8 HE staining

Tissues were taken from mice after execution by anesthesia, placed in 10% neutral formaldehyde fixation for 24 h, gradient ethanol dehydration, xylene fixation, paraffin embedding, sectioning, staining, neutral gum sealing and then observing the lesions under a light microscope.

### 2.9 Statistical analysis

Unsupervised principal component analysis (PCA) was performed using the preomp function within R.^3^ Prior to PCA, the data was scaled to unit variance. For two-group analysis, differential metabolites were determined based on the variable importance in projection (VIP) scores (VIP > 1) and the *p*-value (*p*-value < 0.05, Student’s test). VIP values were extracted from the Orthogonal Partial Least Squares Discriminant Analysis (OPLS-DA) results, which included score plots and permutation plots, generated using the R package MetaboAnalystR. The data underwent log transformation and mean centering before OPLS-DA to ensure data normalization. To prevent overfitting, a permutation test (200 permutations) was performed. Identified metabolites were annotated using the Kyoto Encyclopedia of Genes and Genomes (KEGG) Compound database,^4^ with significantly regulated metabolites were then subjected to metabolite sets enrichment analysis (MSEA), and their significance was determined using hypergeometric test’s *p*-values.

## 3 Results

### 3.1 Evaluation of the A549 model

The growth and progression of tumors in mice were initially assessed. After 21 days post-A549 cell implantation, the tumor model exhibited robust development, with tumor weight showing an increasing trend, as shown in Supplementary Figures 1A,B.

### 3.2 Jidangga-7 regulates the composition of gut microbiota in non-small cell lung cancer model mice

#### 3.2.1 Species relative abundance display

**Figure 1:**
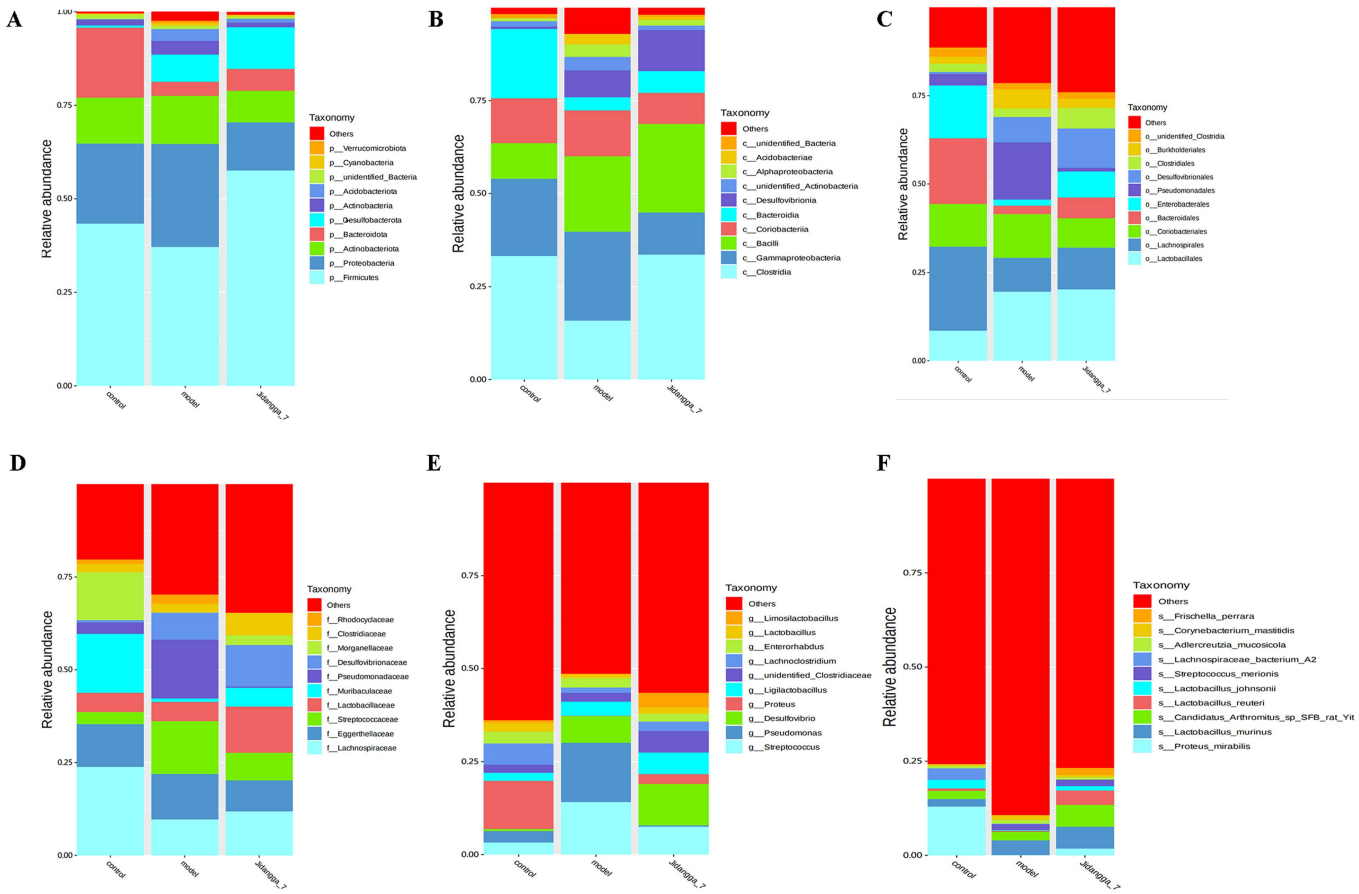
**(A-F)** Stacked bar chart of relative abundance of species in different subgroups at the phylum level based on ASV. Horizontal coordinates are subgroups; vertical coordinates (Relative Abundance) represent relative abundance; others represent the sum of relative abundance of all other phyla in the plot except for these 10 phyla.

Based on the results of species annotation, the top 10 species with the highest abundance in each sample or subgroup at every taxonomic level (phylum, class, order, family, genus, species) were selected to create a bar chart illustrating the relative abundance of species. This visualization allows for the identification of species with higher relative abundance and their proportions at different taxonomic levels in each sample. An example of such a bar chart depicting the relative abundance of species at the gate level is shown in Figures 1A–F.

#### 3.2.2 Sample complexity analysis

Alpha diversity analysis is used to evaluate the diversity of microbial communities within a sample, focusing on within-community richness and diversity. This analysis involves various single- sample diversity metrics such as species cumulative box plots, species diversity curves, and a range of statistically analyzed indices to assess microbial community diversity within each sample. Species dilution curves, also known as rarefaction curves, and rank abundance curves are common representations used to describe the diversity of samples within groups. The results indicate an increase in bacterial abundance in the model group compared to the normal group. Conversely, the administration of Jidangga-7 resulted in a decrease in bacterial abundance compared to the model group.

**Figure 2:**
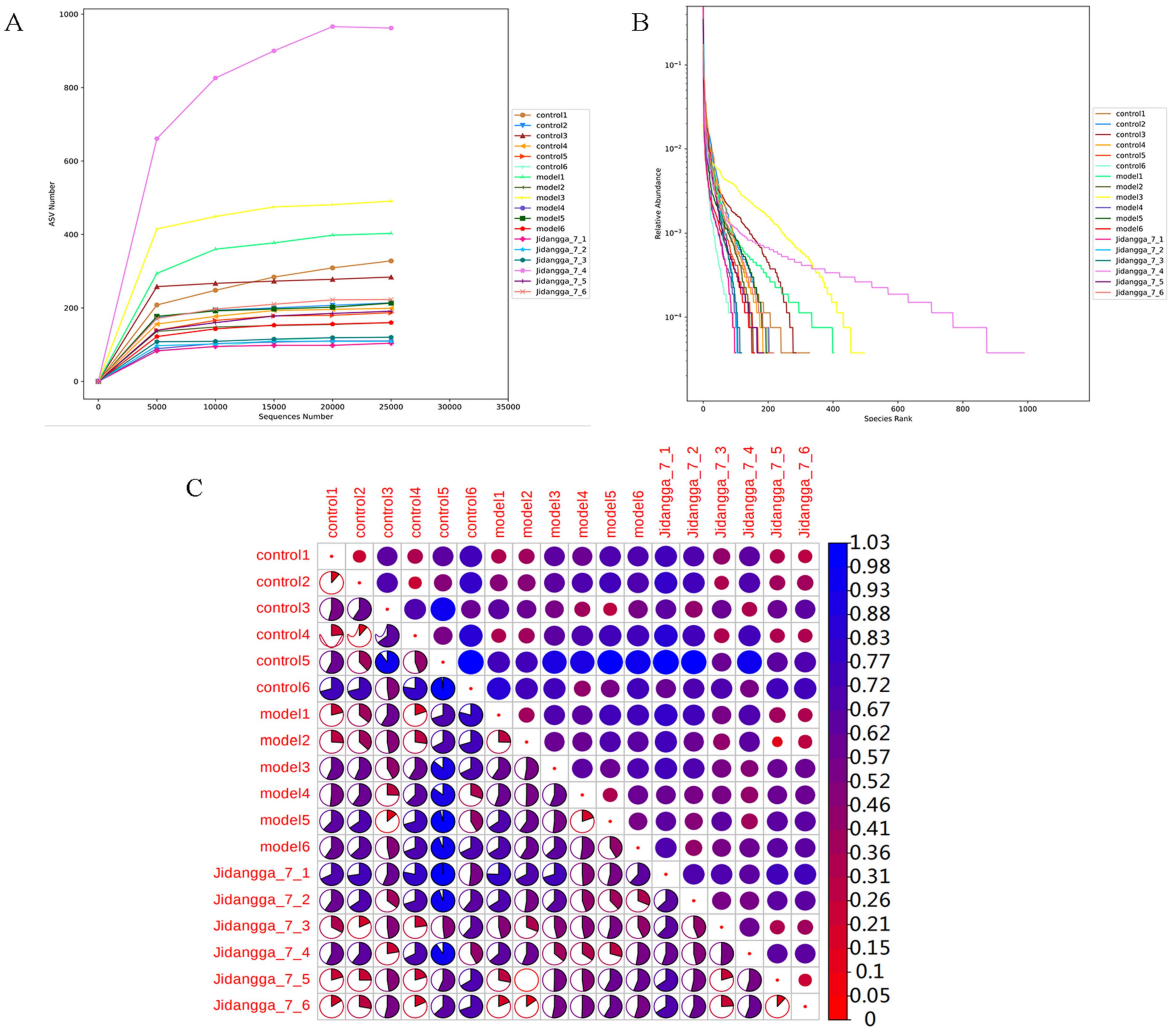
**(A)** Dilution curve of each sample based on ASV, **(B)** ASV-based Rank Abundance curve of each sample, **(C)** ASV-based Beta Diversity Index Heatmap, the circles in the upper triangular box in the figure indicate the beta diversity among samples, the smaller the circle, the redder the color, the smaller the beta diversity value, and the smaller the diversity difference among samples. The smaller the circle, the redder the color, the smaller the beta diversity value, the smaller the diversity difference between the samples.

A dilution curve is constructed by randomly extracting a specific amount of sequencing data from a sample and tallying the number of species they represent, known as the number of amplicon sequence variants (ASVs). The curve can is then plotted with the amount of sequencing data extracted against the corresponding number of species. This curve provides a direct indication of the adequacy of the sequencing data and indirectly reflects the species abundance in the sample. When the curve becomes flat, it suggests that the amount of sequencing data is asymptotically reasonable, meaning that additional data would only produce a marginal increase in the number of new species (ASVs), as detailed in Figure 2A.

The rank clustering curve involves arranging the ASVs in a sample based on their relative abundance or the number of sequences they contain, from highest to lowest. The corresponding rank number of ASVs is then used as the horizontal axis, while the relative abundance of ASVs or the relative percentage of sequences contained in ASVs of the same rank is plotted on the vertical axis. Connecting these points with a polyline yields the rank abundance curve, which visually represents the species present in the sample and provides insights into the richness and evenness of the species. In terms of interpretation, the width of the curve on the horizontal axis reflects species richness, with a wider span indicating higher richness. On the vertical axis, the smoothness of the curve reflects the uniformity of species distribution in the sample, with smoother curves suggesting more even distribution of the species, as visually represented in Figure 2B.

#### 3.2.3 Comparative analysis of diversity

Beta diversity is a comparative analysis of microbial community compositions across different samples. In the Beta diversity analysis, Weighted UniFrac distance and Unweighted UniFrac distance were used to measure the coefficient of variation between two samples, with lower values indicating smaller differences in species between the samples. The Heatmap plotted by Weighted UniFrac distances is shown in Figure 2C. The results indicate a decrease in the abundance of bacteria in the model group compared to the normal group. Meanwhile, the administration of Jidangga-7 led to an increase in bacterial abundance compared to the model group, bringing it closer to the levels observed in the normal group.

**Figure 3:**
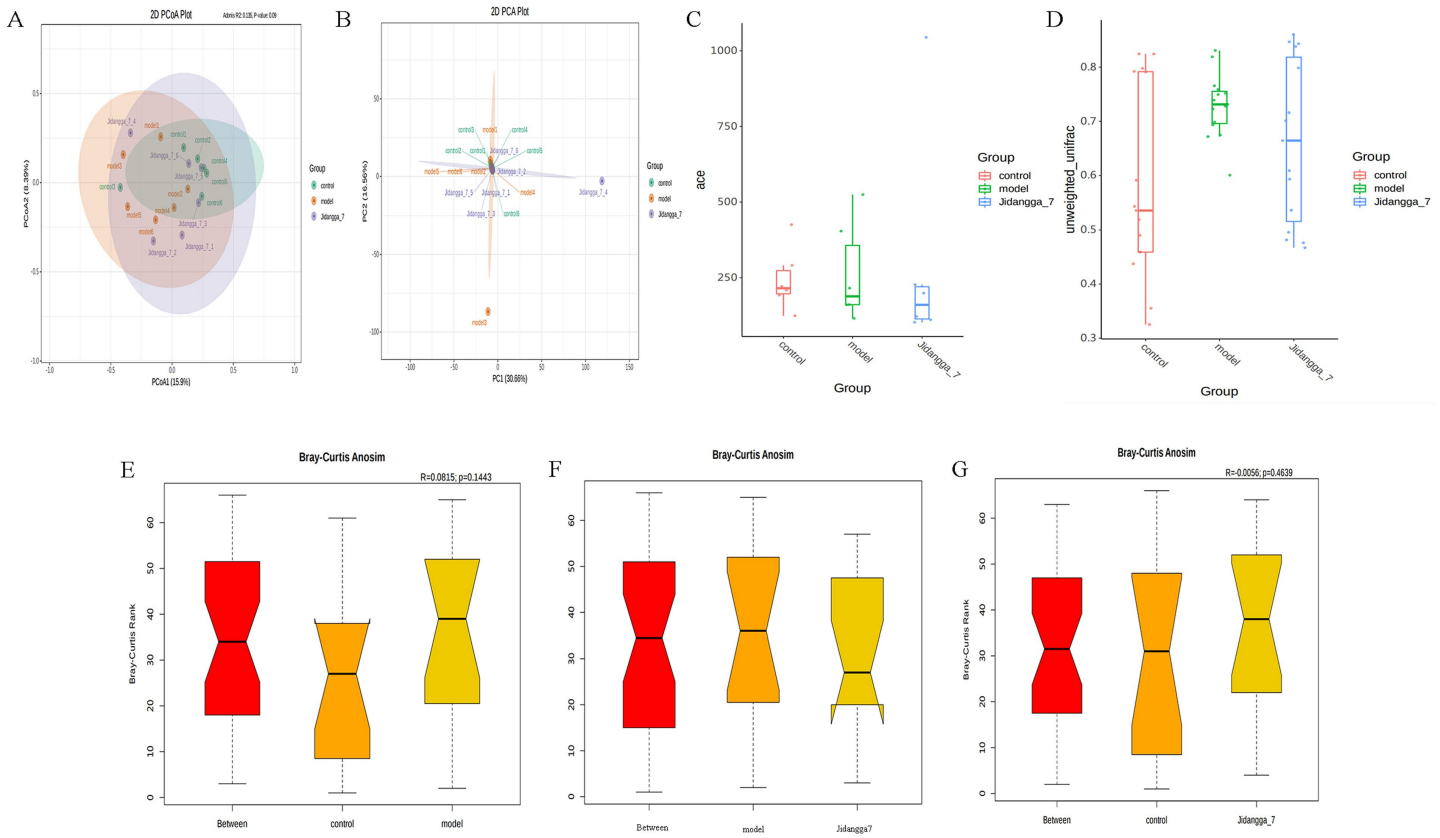
**(A)** Umaeighted Unifrac distance PCoA analysis based on ASV, **(B)** PCA analysis based on ASV, **(C)** box plot of between-group differences in shannon index based on ASV, **(D)** box plot of Unweighted Unifrac Beta diversity based on ASV; **(E–G)** between-group differences in Anosim based on ASV analysis, Vertical coordinate is the rank of the distance between samples, horizontal coordinate: Between is the result between the two groups, the other two are the result within their respective groups.

In Figure 3A, PCoA was utilized to extract the most significant elements and structures from multidimensional data by sorting through a series of eigenvalues and eigenvectors. When samples are closer to each other on the plot, it indicates a greater similarity in species composition. Thus, samples with similar community structures tend to cluster together, while those with distinct community differences are situated farther apart. The results demonstrate noticeable scattering between groups and dispersed aggregation. In Figure 3B, PCA employs variance decomposition based on Euclidean distances to downscale multidimensional data, extracting the most dominant elements and structures. The PCA plot illustrates that the greater the differences in community composition among samples, the more dispersed the distances on the plot become.

#### 3.2.4 Statistical analysis of differences between groups

The significance test for differences in community structure between groups is shown in Figures 3C,D. Anosim analysis, a nonparametric test, was used to assess whether differences between groups were significantly greater than those observed within groups. This analysis helps determine the meaningfulness of groupings. Anosim analysis uses the R vegan package’s anosim function, which conducts significant tests based on the rank order of Bray-Curtis distance values to evaluate differences between groups in Figures 3E–G.

**Figure 4:**
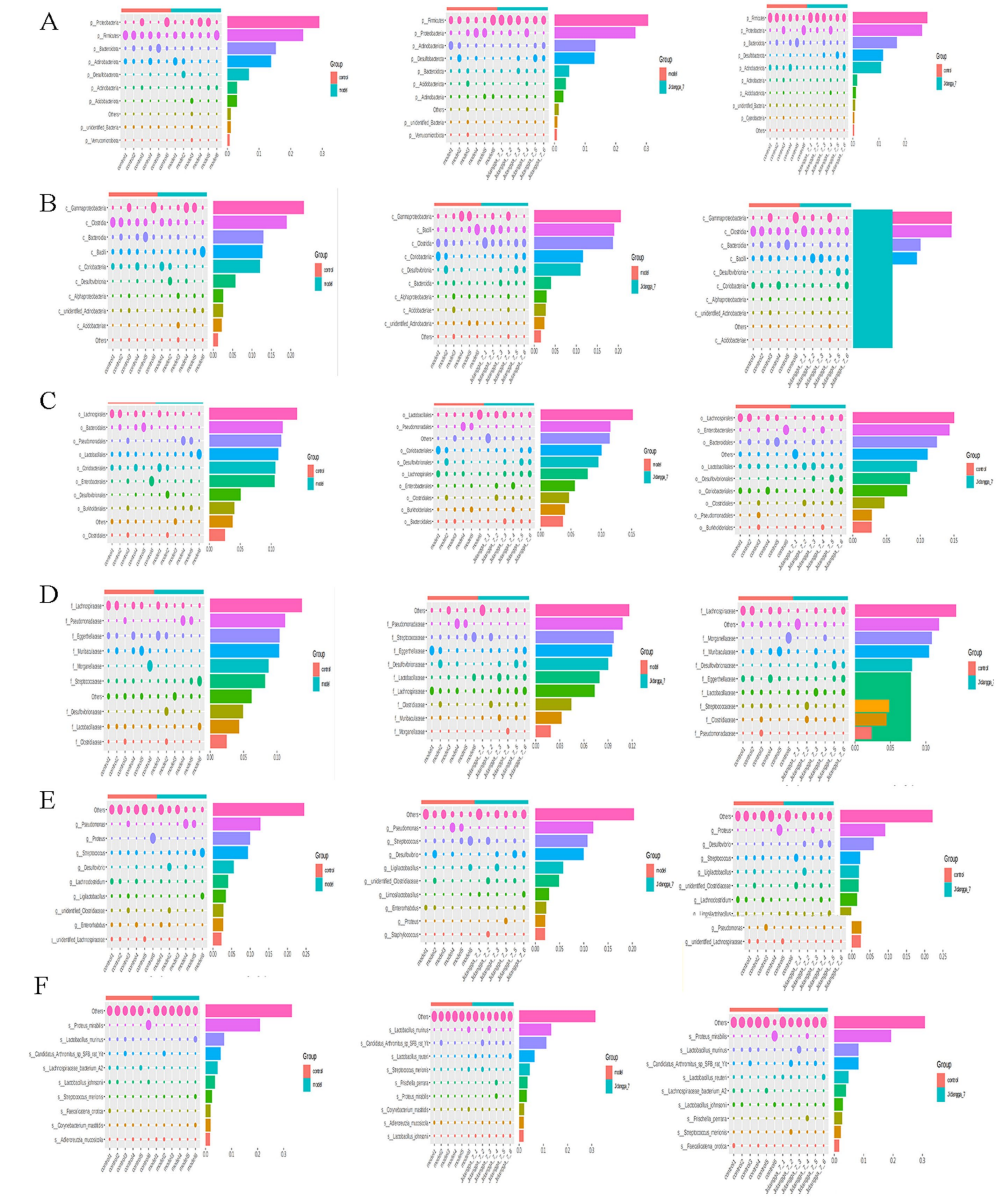
ASV-based simper contribution to difference is plotted with the top 10 contributing species selected by default. The vertical axis represents the species, the horizontal axis is the sample, the bubble size represents the relative abundance of the species, and the Contribution is the contribution of the species to the variability of the two groups. **(A–F)** Phylum, Class, Order, Family, Genus, Species, respectively.

**Figure 5:**
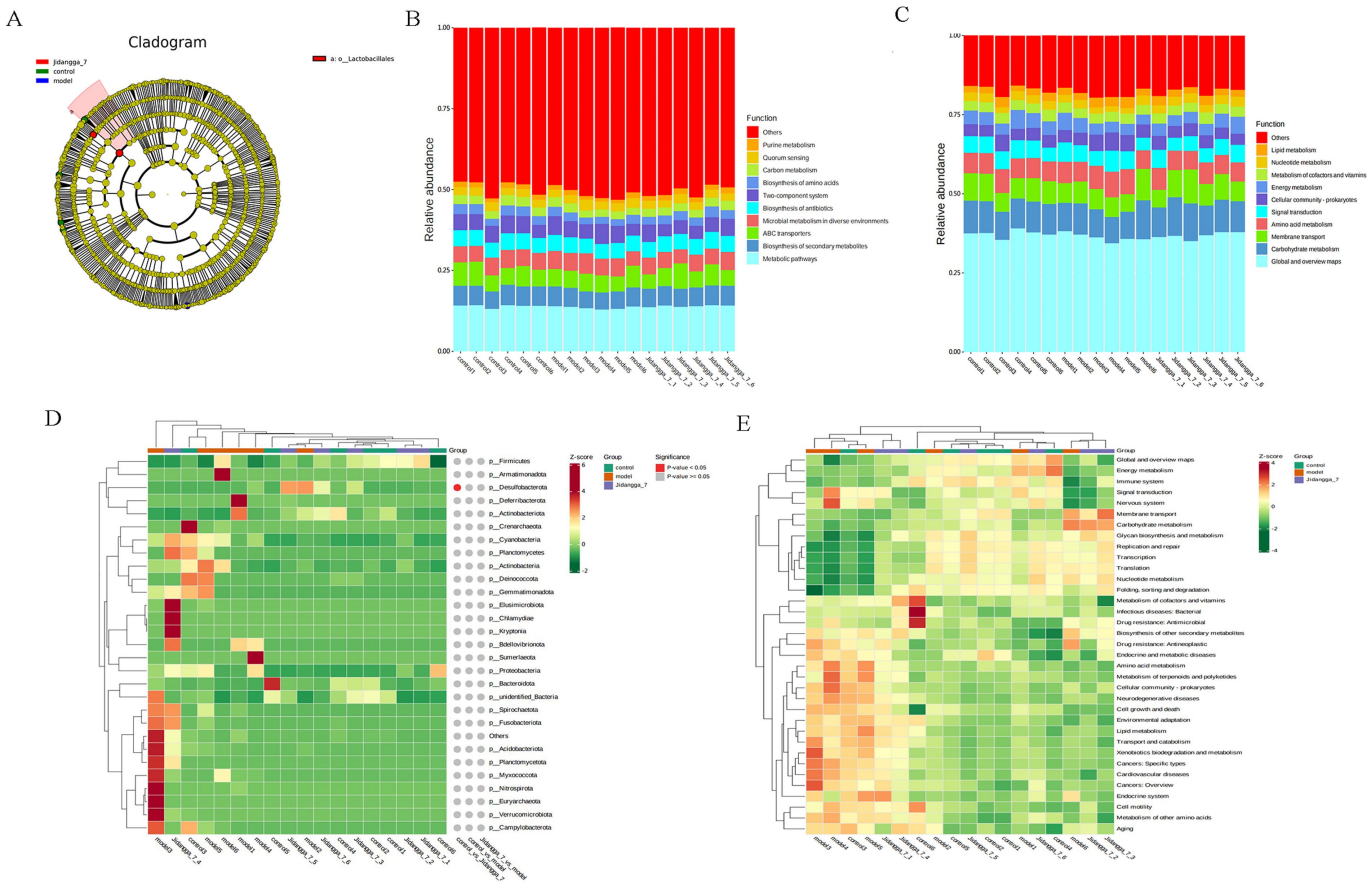
**(A)** ASV-based evolutionary branching map; **(B,C)** Level 1, Level 2 relative abundance histogram; Level 1, Level 2 horizontal clustering heatmap; **(D,E)** Level 1 and Level 2 clustering heat maps.

For projects involving subgroups, conducting in- depth statistical analyses of differences in community structure can provide valuable insights. In this way, species with significant changes in abundance between subgroups can be identified, allowing for the discovery of enriched species across different subgroups. Additionally, comparisons can be made between intra-group differences and inter- group differences to determine the significance of variations in community structure between different subgroups. Simper (similarity percentage) analysis decomposes the Bray-Curtis difference index and quantifies the contribution of each species to the difference between two groups. The results typically highlight the top 10 species and their respective abundances contributing to the difference between the groups. In this study, simper analysis was performed using the simper function of the R software vegan package, and the results are shown in Figures 4A–F. Meanwhile, linear discriminant analysis effect size (LefSe) serves as an analytical tool for identifying and interpreting high- dimensional biomarkers, such as genes, pathways, and taxonomic units. It facilitates comparisons between two or more subgroups, focusing on statistical significance and biological relevance. Therefore, LefSe can identify biomarkers that exhibit statistically significant differences between groups. The evolutionary branching diagram resulting from multiple subgroup comparisons is shown in Figure 5A.

#### 3.2.5 Functional annotation prediction analysis

Based on the database annotation results, the functional information of each sample or subgroup that ranked among the top 10 in terms of maximum abundance at each annotation level was selected. Subsequently, a function relative abundance bar stacking diagram was generated to visually depict functions with higher relative abundance and their proportions at different annotation levels for each sample. For instance, Level 1 and Level 2 relative abundance histograms are illustrated in Figures 5B,C. Furthermore, based on the cumulative abundance of database function annotations across all samples, functions ranking within the top 35 in terms of abundance were selected, along with their abundance information in each sample. A heat map was then created to visualize these functions, clustering them based on differences in functional levels. For example, the Level 1 and Level 2 clustering heat maps are shown in Figures 5D,E.

### 3.3 Jidangga-7 regulates broadly targeted metabolomics in non-small cell lung cancer model mice

#### 3.3.1 Metabolic profiles of each group of samples

**Figure 6:**
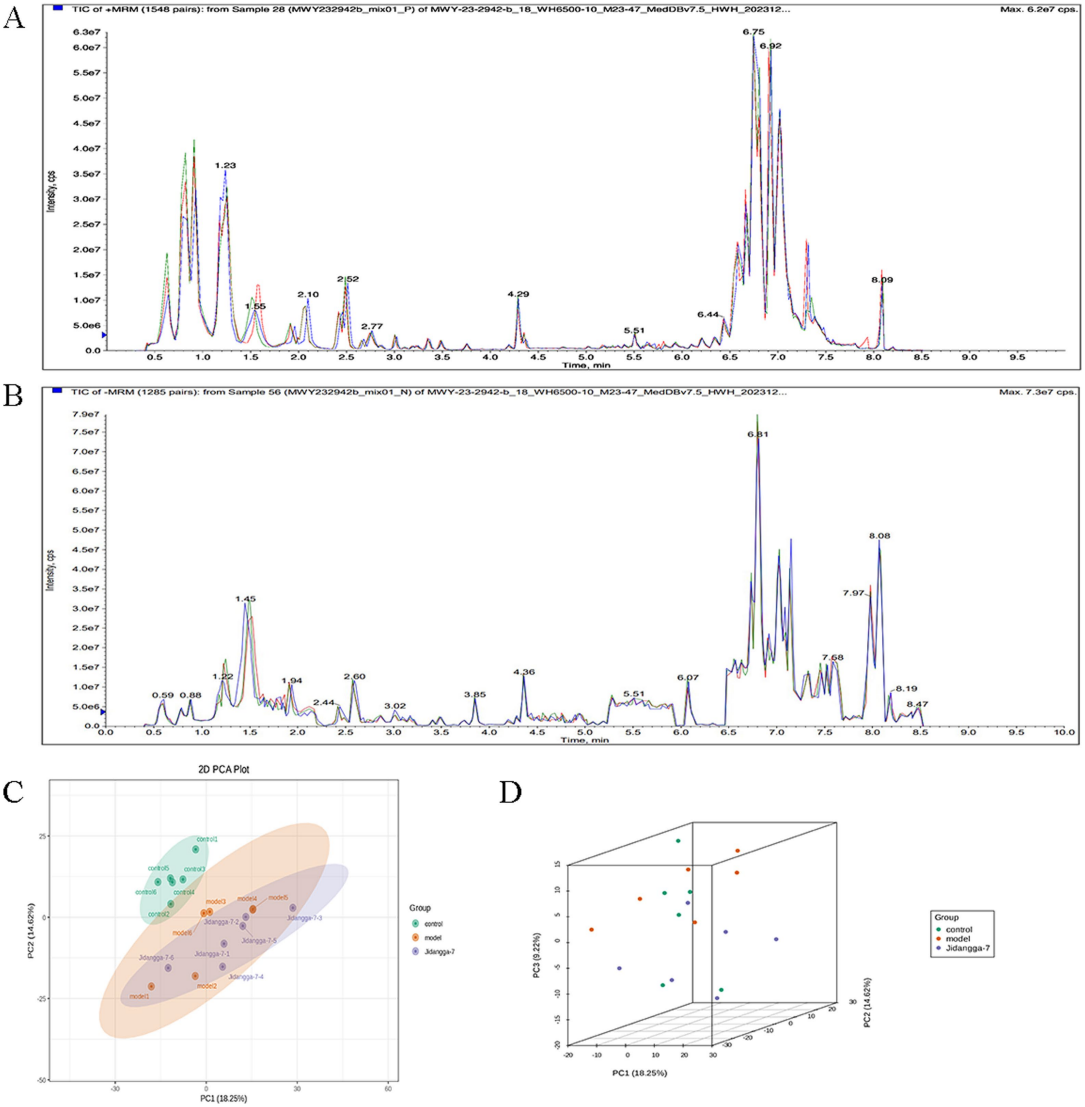
**(A,B)** Multi-peak plots of MRM metabolite detection, **(A)** represents positive ion mode, **(B)** represents negative ion mode; **(C,D)** plot of PCA scores for mass spectrometry data for each group of samples.

The mass spectrometry data underwent processing using Analyst 1.6.3 software. The total ions current (TIC), which represents the sum of intensities of all ions in the mass spectrometry plots at each time point against time, and the multi- peak plots of MRM metabolite detection (ion flow spectrograms of multi-substance extraction, XIC) of the samples are shown in Figures 6A,B. In these plots, the horizontal coordinate represents the retention time (RT) of the metabolite detections, while the vertical coordinate indicates the ion flow intensity of ion detection, measured in counts per second (CPS).

#### 3.3.2 Results of principal component analysis for each group

To get a preliminary knowledge of the overall metabolite differences between groups of samples and the degree of variability between samples within groups, the samples were subjected to principal component analysis (PCA). Whether or not metabolite groups varied within groups as revealed by the PCA data, there is an observed pattern of metabolite separation across groups (Figures 6C,D). Samples clustered together in each group indicate that there is little variation between the groups; if the samples are farther apart, it means that there is more variability between the samples.

#### 3.3.3 Dynamic analysis of metabolite content differences

**Figure 7:**
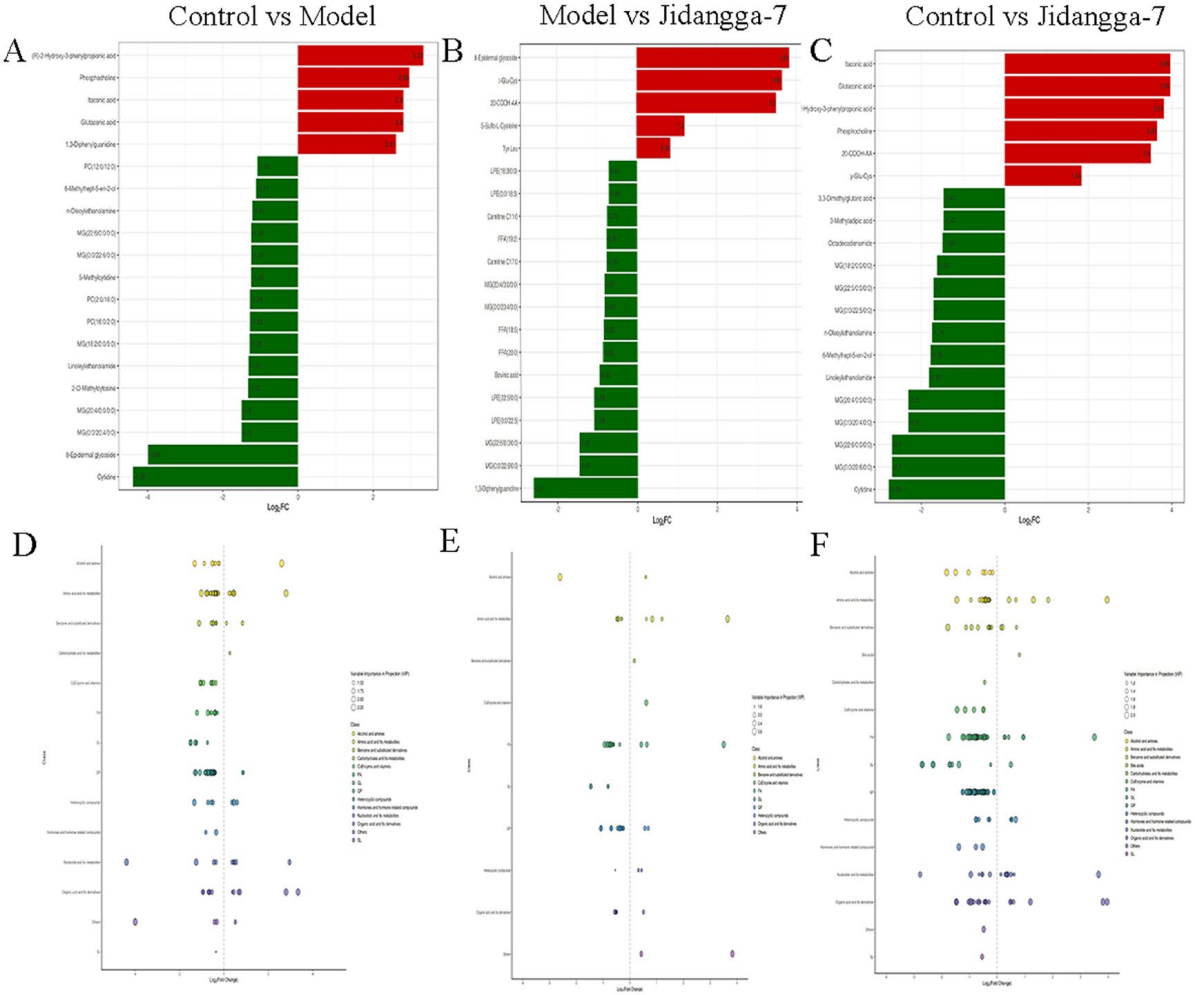
**(A–C)** Differential multiplicity bar chart, red represents up-regulation of metabolite content, green represents doxm-regulation of metabolite content; **(D–F)** differential metabolite scatter plot.

To provide a clearer and more intuitive representation of the overall metabolic differences, the f old change (FC) values of metabolites in the comparison group were calculated. Subsequently, metabolite contents were ranked from smallest to largest according to FC values. The dynamic distribution of metabolite content differences was plotted, with the top 20 metabolites labeled for up-regulation and down- regulation, as illustrated in Figures 7A–C. Up-regulated metabolites include: ltaconic acid, glutaconic acid, phosphocholine, glu-cys. Down-regulated metabolites include: 3, 3-Dimethyglutaric acid, 3-Methlgutaric acid MG(20:4/0:0/0:0).

#### 3.3.4 Differential metabolite correlation analysis

**Figure 8:**
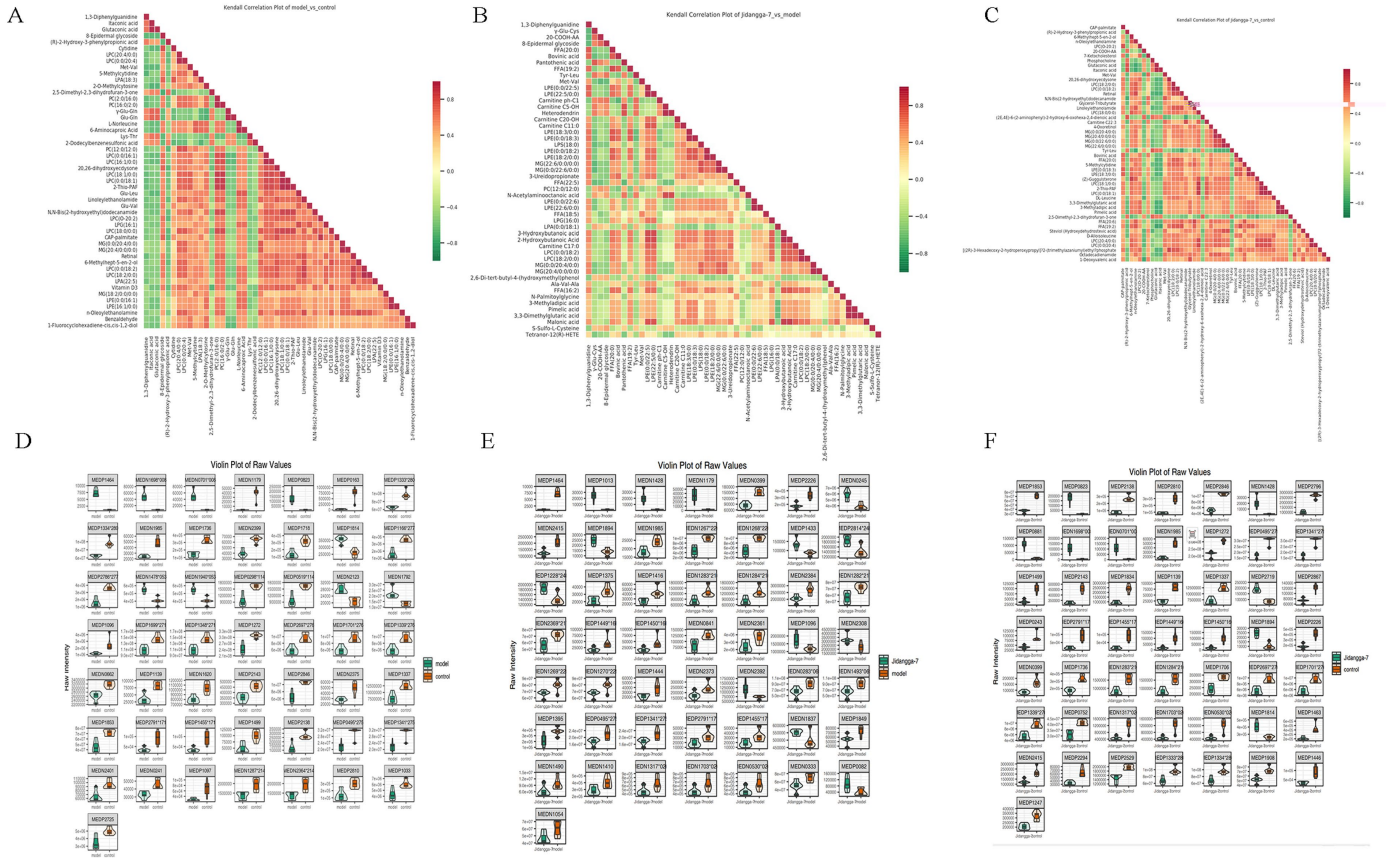
**(A–C)** Differential metabolite correlation heatmap, horizontal is the name of the differential metabolite, vertical is the name of the differential metabolite, red indicates a stronger positive correlation, green indicates a stronger negative correlation, and the deeper the color represents the greater the absolute value of correlation coefficients between the samples; **(D–F)** differential metabolite violin plot, horizontal coordinate is group, vertical coordinate is relative content of differential metabolite (original peak area).

Differential metabolite scatter plots are mainly used to illustrate the relative content variances of different classes of substances in two groups of samples, showcasing the differential metabolites identified within each comparison group, as presented in Figures 7D–F. Various metabolites exhibit synergistic or mutually exclusive relationships, and correlation analyses aid in assessing the degree of metabolic closeness (metabolic proximities) between significant differential metabolites. This facilitates a deeper understanding of the inter-regulatory relationship between metabolites during biological state changes. Pearson’s correlation analysis method was employed to analyze the correlation among the identified differential metabolites based on the screening criteria, with the results shown in Figures 8A–C. Additionally, the violin plot, which combines features of both box- and- line plots and density plots, is used to visualize data distribution and its probability density. The central box represents the interquartile range, while the thin black line extending from it represents the 95% confidence interval. The median is denoted by the black horizontal line in the center, while the outer shape indicates the distribution density of the data, as detailed in Figures 8D–F.

#### 3.3.5 Cluster analysis of Kyoto Encyclopedia of Genes and Genomes pathway differential metabolites

**Figure 9:**
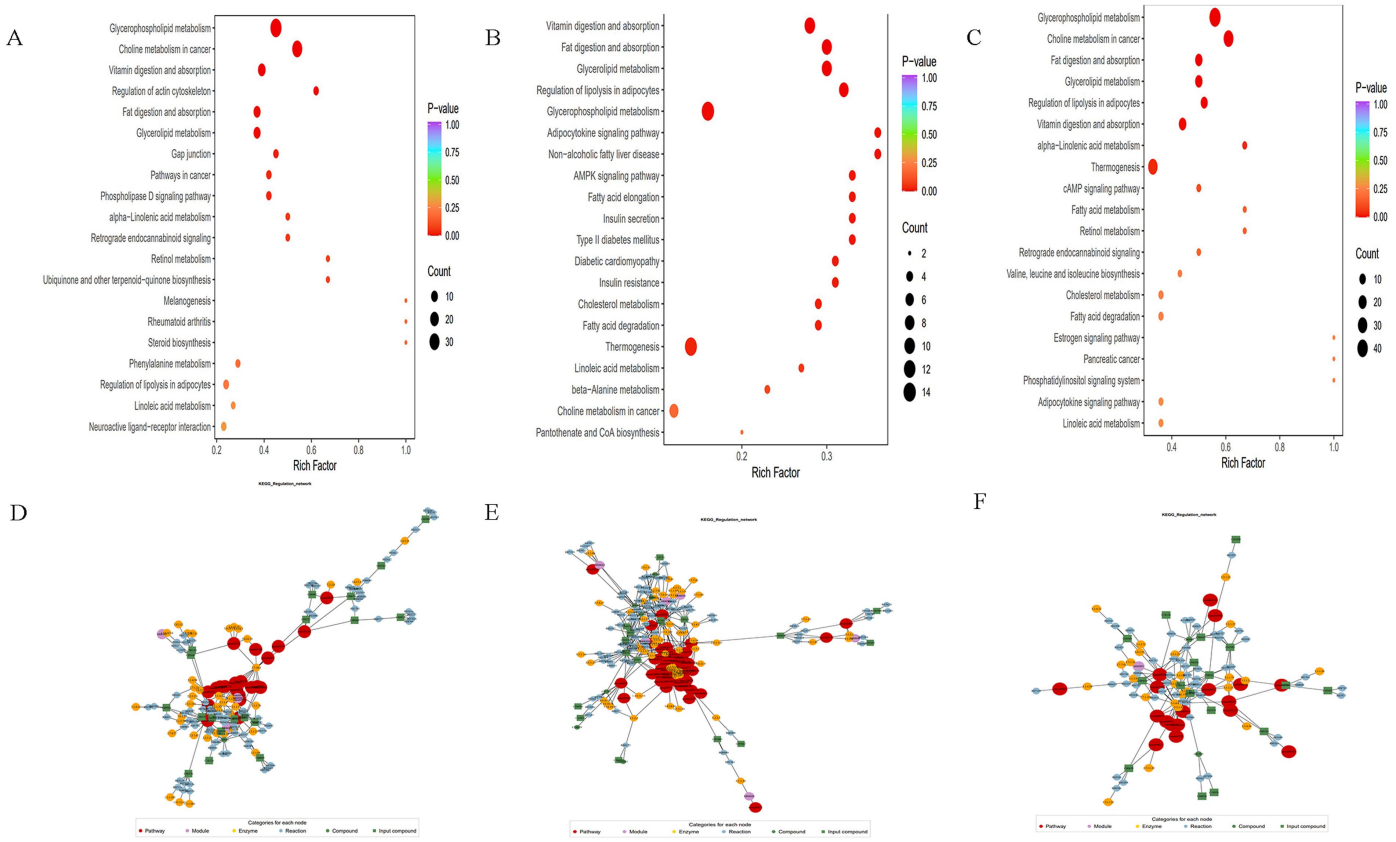
**(A–C)** Differential metabolite pathway enrichment map, the color of the dots reflects the *p*-value size, the redder it is, the more significant the enrichment is. The size of the dots represents the number of different metabolites enriched; **(D–F)** differential metabolite regulatory network diagram, red dots represent a metabolic pathway, yellow dots represent information of a substance related to regulatory enzymes, green dots represent background substances of a metabolic pathway, purple dots represent information of the molecular module of a class of substances, blue dots represent information of a substance’s chemical interactions, and green squares represent different metabolites obtained from the present comparisons. The green squares represent the differential metabolites obtained in this comparison.

Using the KEGG annotation data of the identified differential metabolites based on the screening criteria. Clustering analysis was then conducted on all differential metabolites within these pathways. If a pathway contained fewer than five differential metabolites, it was excluded from display, as illustrated in Figures 9A–C. Furthermore, the analysis of KEGG functional annotations and disease correlation highlighted impacts on cofactors and vitamins, bacterial, antimicrobial, and xenobiotics biodegradation and metabolism. Subsequently, after obtaining matching information for the differential metabolites, pathway search and regulatory interactions network analysis were performed according to the KEGG database of the corresponding species, which was displayed as a network plot. This analysis was specifically performed for human, mouse, and rat plots, as shown in Figures 9D,E.

### 3.4 HE staining

**Figure 10:**
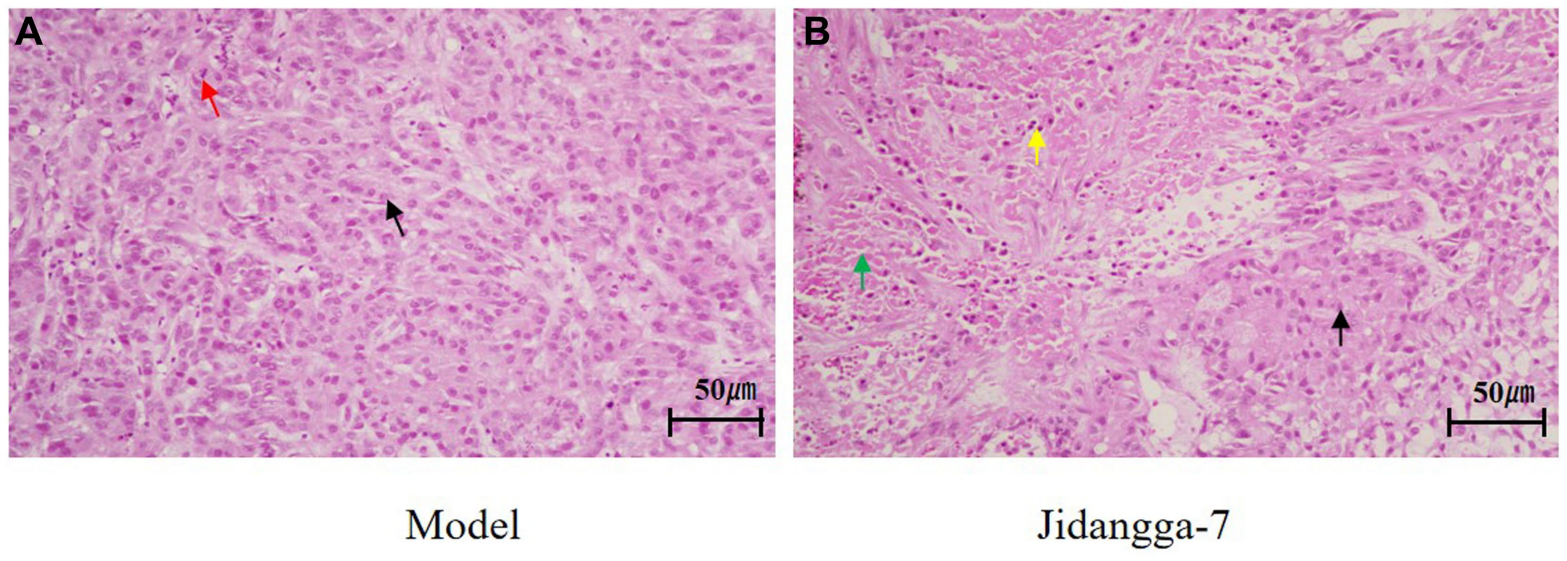
Tumor HE staining results, **(A)** the model groups, **(B)** the treatment group (x200).

The results of tumor histopathological staining sections transplanted with A549 tumor cells in nude mice were observed. In the model group, the tumor structure was normal, the tumor cells were regular in morphology, large in number, orderly in arrangement, and did not show obvious degeneration, as shown by the black arrowheads; suggesting that the tumor was growing, as indicated by the red arrowheads; in some regions of the therapy group, a significant percentage of tumor cells were necrotic and degraded, and the green arrowheads showed that the cytoskeletons’ nuclei had disintegrated or vanished; with the nuclei of the cytoskeleton crumpled up and deeply stained and the cytoplasm of the cytoplasm red stained, as shown by the yellow arrowheads The overall structure of the tumor was loose and the interstitial space was enlarged, and the normal tumor cells were shown by black arrows, shown in Figure 10.

## 4 Discussion

In Mongolian medicine, Pi disease is caused by the decay of the stomach fire and the gradual weakening of the heat energy distributed in all parts of the human body, and the destruction of the relative balance relationship with the seven elements (Dietary essences, bone, blood, marrow, muscles, sperm, adipose), resulting in dysfunction, and the blood and essence in the liver cannot be biochemically and become turbid, and then the evil blood and XiriUsu surge, staying in the weak place of a certain viscera, and the power of Heyi coalesces into a Pi (Cesurongzhabu, 2011). The body is an organic whole composed of internal organs, tissues and organs, and each component is interconnected, mutually supportive and mutually influential. Mongolian medicine Jidangga-7 is warm, hot, light and sharp, and has cold, heavy, dull and solid temperaments respectively, so as to play a role in the treatment of Pi. The results showed that Jidangga-7 had an inhibitory tendency to inhibit the growth of human non-small cell lung cancer A549 cells in subcutaneous xenografts in nude mice (Supplementary Figure 1B). The morphological observation results of HE staining showed that compared with the model group, the number of tumor cells in the Jidangga-7 group was significantly reduced, the arrangement was sparse and disordered, the cells were significantly atrophied, the nuclei were solid and unclear, and the loose connective tissue was significantly increased. Taken together, the above results showed that Jidanga-7 had a significant inhibitory effect on the growth of lung adenocarcinoma.

Lung cancer is the most prevalent and fatal malignancy in both China and globally, with annual lung cancer-related deaths in China exceeding the combined total of colorectal, breast, and prostate cancers (Torre et al., 2016; Miller et al., 2020). Therefore, effective lung cancer treatment remains a top priority in clinical oncology. To increase therapy effectiveness and extend the lifespan of patients with non-small cell lung cancer, non-invasive biomarkers with high sensitivity and specificity must be developed. Several studies (Zheng et al., 2020; Nagasaka et al., 2020; Lim et al., 2021) have shown that NSCLC patients have increased levels of pathogenic bacteria, such as *Actinobacteria*, *Streptococcus*, and *Clostridium*, along with decreased levels of probiotic bacteria such as *Bifidobacteria*, *Lactobacillus*, *Enterococcus faecalis*, and *ruminal Bacillus* in their gut microbiota compared to healthy individuals. In conclusion, the following traits describe the gut microbes that are typical of lung cancer patients: *(1) a decline in probiotics; (2) an increase in pathogenic bacteria; (3) a rise in species belonging to the Mycobacterium phylum and a fall in species belonging to the Mycobacterium thick-walled; however, a reduction in the ratio of Mycobacterium thick-walled to the Mycobacterium phylum may result in lower levels of short-chain fatty acids in the blood* (Jin et al., 2019; Pinato et al., 2019). While short-chain fatty acids are crucial for host systemic immunity and systemic inflammation (Pinato et al., 2019; Wypych et al., 2019), they can also cause lung cancer cells to undergo apoptosis and cell cycle arrest (Dang and Marsland, 2019). This alteration promotes dysbiosis in intestinal homeostasis, suggesting a potential link between gut flora composition and NSCLC.

### 4.1 Relative abundance of intestinal flora species and abundance clustering analysis

The analysis of relative abundance of Level 1 and Level 2 revealed that at Level 1, microbial metabolism in diverse environments, the two-component system, biosynthesis of antibiotics, and carbon metabolism were predominant. Similarly, Level 2 also emphasized global and overview maps, carbohydrate metabolism, membrane transport, and cellular community-prokaryotes.

### 4.2 Analysis of differences between groups and functional annotation

Analyzing the disparities in Alpha and Beta diversity indices between groups revealed significant distinctions. The examination of community structure disparities indicated notable differences among colonies between groups. Sipmer analysis quantified the contribution of each species to the differences between the two groups, highlighting the top 10 species and their abundance influencing the disparities. At the class level, the Jidangga-7- treated group exhibited regulatory effects on Gammaproteobacteria, Bacilli, *Desulfovovoviridae*, *Desulfoviridaceae*, and *Desulfovibrionia*. Gammaproteobacteria are a significant group of microorganisms in the human gut that support immunomodulation, nutrient absorption, and intestinal microecological balance. Gammaproteobacteria can generate advantageous metabolites like short-chain fatty acids, which are crucial for preserving intestinal microecological balance and advancing health (Saksham et al., 2023). It is also involved in the regulation of intestinal mucosal immunity and fights pathogen invasion by stimulating the host immune response (Saksham et al., 2023). A study has shown an association between the Bacillus order (Bacilli class) and lung cancer (Dean et al., 2021). Similarly, at the family level, the Jidangga-7-treated group demonstrated regulatory effects on *Desulfovibrionaceae*, *Lachnospiraceae*, and *Eggerthellaceae* compared to the model group. Improved metabolism of Desulfovibrionaceae was discovered to improve the frequency of CD8 + T cells, boost interferon-stimulated gene (ISG) expression, and together enhance mice’s anti-PD-1 ability to have anti-tumor effects (Jaeho et al., 2023). Lachnospiraceae family bacteria are thought to inhibit colorectal cancer and fight tumors by promoting immune surveillance functions (Xusheng et al., 2023).

#### 4.2.1 Broadly targeted metabolomics in non-small cell lung cancer model mice

Extensively targeted metabolomics integrates the broad range of non-targeted metabolomics with the accuracy of targeted metabolomics, offering high-throughput, ultra-sensitive, wide- coverage, and quasi- qualitative and quantitative analysis capabilities. In the present study, PCA and S-PLOT analyses revealed a clear distinction between the groups, with the Jidangga-7 group closely resembling the normal group. The KEGG analysis of differential metabolite regulatory networks showed that Jidangga-7 could regulate the gut microbiota in NSCLC through various pathways, aligning with the concept of traditional Chinese medicine’s multi-target and multi- pathway regulation approach. Furthermore, the heat map of differential metabolite clustering illustrated that Jidangga-7 exhibited regulatory effects on metabolites such as carnitine ph-Cl, N-acetylaminooctanoic acid, andN’-methyl-2pyridone-5- carboxamide, compared to the model group. Research has indicated that metabolically active molecules associated with gut microbiota influence host metabolism, impacting disease progression (Guzior and Quinn, 2021). Imbalances in intestinal metabolism lead to the production of a variety of toxins and promote the generation of free radicals and reactive oxygen species, which can exert carcinogenic effects through multiple pathways. For example, the toxin produced by *Pseudomonas fragilis* acts as a virulence factor that up- regulates the bacterial polyamine catabolic pathway and generates reactive oxygen species (Dalal et al., 2021). KEGG enrichment analysis showed that Jidangga-7 regulated pathways such as glycerophospholipid metabolism, vitamin digestion and absorption, thermogenesis, cholineogenesis, and cholinesterase, compared to the model group (Wang et al., 2020; Wang et al., 2023). These pathways play crucial roles in regulating the intestinal flora in non- small cell carcinoma. However, further research is necessary to explore additional metabolic pathways in depth.

Although this study revealed the association between lung cancer and intestinal flora to a certain extent, and also showed that the increase of beneficial flora after the application of drug treatment could improve the changes of intestinal flora in mice with lung cancer, further study on the mechanism of action is still needed; secondly, the study was only applied to animal experiments and was not confirmed in clinical studies, and the next study should be focused on the clinical study to observe the clinical efficacy of patients with lung cancer and the changes of intestinal. The next study should focus on clinical research to observe the clinical efficacy and intestinal flora changes in lung cancer patients, so as to provide new valuable findings and research basis for clinical application.

## 5 Conclusion

Several studies have highlighted the potential of regulating gut flora homeostasis as a novel approach to treating NSCLC patients, aiming to improve treatment outcomes by manipulating gut bacteria. Gut flora offers a promising avenue for exploring new strategies in the development, progression, diagnosis, treatment, and prognosis of NSCLC. However, its clinical application is still challenged by factors related to age, gender, disease status and environmental influences, which can significantly impact the composition of the intestinal microbiota, varying among individuals. Traditional Chinese compound preparations, with their multi- molecular targeting approach, offer a means to integrate small molecule compounds to collectively influence the intestinal flora and improve the gut environment. In the future, a comprehensive understanding of the mechanisms underlying the interaction between bacterial flora, host cells, and tumor cells is needed. This understanding is expected to guide the development of more effective treatments for NSCLC and ultimately improve patient prognosis.

## Data Availability

The datasets presented in this study can be found in online repositories. The names of the repository/repositories and accession number(s) can be found in the article/Supplementary material.

## Footnotes

^1^ https://sciex.com.cn/

^2^ https://sciex.com/

^3^ www.r-project.org

^4^ http://www.kegg.jp/kegg/compound/
