# Supplementary figures and images for: Jidangga-7 ameliorates non-small cell lung cancer by regulating gut microbiota function
Source: Front Microbiol. 2025 Mar 5;16:1516685. doi: 10.3389/fmicb.2025.1516685 (PMC11919877; doi:10.3389/fmicb.2025.1516685)

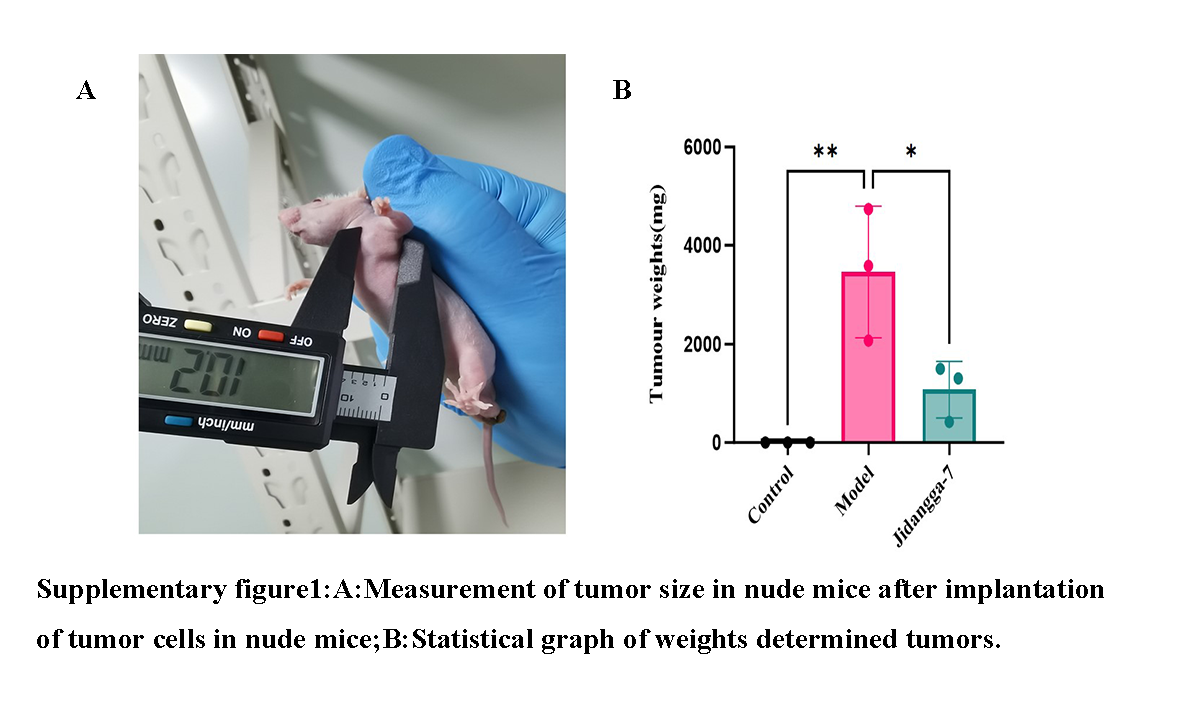

Supplement: Supplementary file 1 [file Image_1.TIF]
